# U. S. V. M. A.

**Published:** 1896-07

**Authors:** 


### U. S. V. M. A.

Dr. L. A. Merillat, Secretary of McKillip Veterinary College, Chicago, will present a paper at Buffalo on "Veterinary Dental Surgery."

Dr. John W. Adams, of the University of Pennsylvania, will discuss the position of the colleges in relation to their support and direction of schools of farriery.

The Association of Faculties will probably hold their annual gathering a day or two in advance of the U.S.V.M.A. meeting. Secretary Adams is in active communication with the heads and members of all the veterinary faculties in North America, and a strong and enthusiastic meeting is assured.

Prof. Schwarzkopf has lost none of his keen interest in veterinary education in America, and will be an earnest and forceful advocate at the Buffalo meeting of higher veterinary education.

There will be a week of good things at Buffalo and the literary attractions will be a feast for everyone to indulge in. Let there be but few stay-at-homes.

The Northwest will be heard in the convention, and the ever-important subject of the "Nail-wounds of the Foot" will be presented by Dr. S. B. Nelson, of Pullman, Washington. The subject will be opened in discussion by Prof. Zuill, of Philadelphia.

The members will be regaled by the consideration of "Serum-therapy in Hog-cholera," by Dr. A. F. Peters, of the Nebraska experiment station.

The completion of a long line of experiments by Prof. M. H. Reynolds, of Minnesota, in determining the relative value of "cathartics," will be further reported.

A paper on "Dairy Inspection," by Dr. Fred Braginoton, of Indiana, has been added to the list of subjects.

Prof. Grange, of Michigan, and Prof. White, of Ohio, will both offer papers at this meeting; the subjects will be announced in the August number of the Journal.

The programme for Buffalo will present the most widely distributed list of papers, geographically, ever presented at a single meeting in the Association's history.

Drs. Dalrymple, of Louisiana; Bird, of Montana; Mayo, of Kansas; Hinebauch, of North Dakota, are also expected to contribute papers for this meeting.

Reports of committees and papers will have a time allotted to each by the *Comitia Minora*, an outline of which will be given in the August number.

*On to Buffalo.* The Empire State sends greetings and a welcome to every veterinarian to come and bask in her hospitality.

Chairmen Clement and Harger, of the Intelligence, Education, and Disease Committees, are rapidly maturing their plans for a complete survey of the field. Prompt replies to their requests are earnestly urged upon all the members.

Wake up, State Secretaries, there is much for you to do; you have accepted the responsibilities, and Chief Secretary Stewart is anxious about your work.

Treasurer Robertson says he will be there with his little boomlet on hand, bent for the Pacific coast, if it is not too warm to urge it.

State Secretary Rhoads has in preparation a letter to members of the profession in Pennsylvania urging their attendance at Buffalo, and calling upon the profession to more strongly ally themselves with the parent organization.

A party of Philadelphians hope to arrange for a ten days' outing in connection with the annual meeting at Buffalo.

Reports from the Eastern States indicate a large attendance from the New England district.

A review of the work and province of State veterinary examining boards will be a feature of the meeting at Buffalo. This subject should merit the attention of the heads of colleges.

There will be no prizes offered this year by the Association for papers, owing to a deficit in the treasury.

The Publication Committee will be heard from, and it is rumored that they will speak in no uncertain tones. It is to be hoped that there will be few delinquents on the dues list, so that the falling stones will have but few targets to spend their force upon.

Messrs. Osgood, Lyman, Salmon, Pearson, Trumbower, and others will discuss tuberculosis in its present and future important aspects.

The work of the Master Horseshoers' Association, in encouraging the establishment of schools of farriery for the better education of their craftsmen, will be presented for the information and consideration of the Convention.

The Schuylkill Valley Veterinary Medical Association sends as her delegate their President, Dr. James W. Sallade, one of the Keystone State's most active veterinarians.

President Ridge, of the Pennsylvania State Veterinary Medical Association, has named the ever-faithful Dr. Foelker, of Allentown, as one of the delegates; associated with him are Dr. George B. Jobson, of Franklin, and Dr. James T. McAnulty, of Philadelphia, one of the most ardent and efficient workers in the national and local master horseshoers' organizations.

Municipal legislation as it applies to milk- and meat-inspection will come in for a goodly share of attention. A generous invitation is extended to all veterinarians who hold these positions to come and contribute their views.

The colleges of our country will come in for a generous meed of praise for the great advances they have made in extending higher veterinary education over the entire land.

Secretary Morton will be appealed to for the most generous indulgence, with due regard to the public service, in granting leave of absence to the veterinarians in the service, that they may contribute to the discussion of the many aspects of national, State, and inter-State inspection of food-products at Buffalo.

It is to be hoped that the veteran member, Prof. Liautard, will return from sunny France in time for daily attendance on the sessions of the convention.

Nebraska veterinarians are fully alive to the importance of the convention at Buffalo, and Secretary Peters hopes to outdo at Buffalo the grand work done in the line of attendance by Minnesota at Des Moines.

Buffalo will afford no excuse for non-attendance of the members in the lake States; facilities favor these representatives greatly at this time.

New York City will send to the "Queen City of the Lakes" some new faces of old members, whose renewed interest in Association affairs will be gratefully appreciated by the present officers.

State Secretary Rhoads, of Pennsylvania, has identified himself with the Masonic Order in the Keystone State; but he says this will only intensify his work in the interest of Buffalo.

Secretary Stewart's gentle reminder that you are in arrears, Mr. Member, comes to us couched in the softest language and makes one feel a little uncomfortable and chagrined at our dereliction in this direction. He fittingly says that the work already done by the National Association for the profession is of such worth and magnitude that, had it cost ten times the amount expended, it would still have been wisely used means.

Treasurer Robertson sighs for the days that are gone when the surplus in the treasury was of such magnitude as to cost hours of anxiety as to its safe-keeping. He finds himself now without an occupation and yearns for something to do. Pay up your back dues, fellow-members.

A large number of certificates of membership was issued in June by Secretary Stewart. There are more to follow this month.

Chairman Hinkley, of the local committee of arrangements, says nothing will make him happier than the largest attendance that has occurred at any of our meetings for years. State Secretaries, take notice.

A leading Eastern veterinarian says he will not occupy a post of distinction alongside the driver in attending the sessions from his hotel, *á la* Des Moines. It is to be hoped that it will not be so warm, for he says he would rather walk.

Chairman James Robertson, of Chicago, of the Master Horseshoers' Association, will be at Buffalo, and report the educational progress of their State and local schools of instruction in farriery. Dr. McAnulty, of Philadelphia, another member of the committee, will also be present to supplement the report of the chairman.

Dr. J. F. Winchester, of Lawrence, Mass., will present a paper on "Diphtheria" at the coming convention. Among those who have been assigned places for the discussion of this paper will be Prof. James L. Robertson, of New York City, and W. L. Zuill, of Philadelphia.

The Genesee Hotel will be made the headquarters of the convention during our stay in Buffalo. Ample accommodations for the members and delegates will be provided for in the way of rooms for committee meetings and kindred associations.

The New York State Association will convene at Buffalo on the 4th and 5th of September, when a very attractive programme will be presented, to which all the delegates and members of the U. S. V. M. A. will be accorded a warm welcome. Among the interesting papers to be presented for consideration are: "Veterinary Education," by Dr. Thomas Giffen, of New York City; "Navicular Disease in Horses," by Dr. George H. Berns, of Brooklyn; "Veterinary Jurisprudence," by Dr. J. P. Thomson; "Bacteria in Milk," by Dr. Wilson Huff, of Rome; "Veterinarians as Sanitarians," by Dr. Claude D. Morris, of Pawling. A number of interesting operations will be performed in connection with the State meeting at the hospitals of the local veterinarians.

Buffalo has 150 miles of street railways; 26 railways enter the city; 250 trains daily arrive and depart on these roads; nearly 1800 miles of asphalted streets; 17 miles of park driveways.

